# Prevention of Corneal Neovascularization; a Preliminary Experimental Study in Rabbits

**Published:** 2020-01-01

**Authors:** Ali Kasiri, Mohammad Sadegh Mirdehghan, Fereydoun Farrahi, Farshad Ostadian, Mostafa Feghhi, Mehdi Reza Ghomi, Aram Mohammad Jafari, Atefeh Mahdian Rad, Niusha Kasiri

**Affiliations:** 1 Department of Ophthalmology, Faculty of Medicine, Infectious Ophthalmic Research Center, Ahvaz Jundishapur University of Medical Sciences, Ahvaz, Iran; 2 Medical Student, Ahvaz Jundishapur University of Medical Sciences Ahvaz, Iran

**Keywords:** Corneal Neovascularization, Propranolol, Timolol, Bevacizumab, Betamethasone

## Abstract

The purpose of this study was to compare the effects of propranolol, timolol and bevacizumab with betamethasone to prevent corneal neovascularization (CNV) in rabbits. This study was performed on 28 male rabbits. CNV was induced by three 7-0 silk sutures 2 mm long and 1 mm distal to the limbus. Animals were randomly divided into 4 groups of propranolol + betamethasone, timolol + betamethasone and bevacizumab + betamethasone and betamethasone alone. Eye drops were started from the first day of study. On 7^th^, 14^th^, 21^st^, 28^th^, 35^th^ and 42^nd^ days, vascular progression, time of neovascularization and vascular area were evaluated and compared with the control group (betamethasone alone). There was a significant reduction in the area of ​​neovascularization in the timolol and bevacizumab groups compared to the control group (P-value = 0.05, P=0.047, respectively). Also, regarding vascular progression, there was a significant decrease in the timolol and bevacizumab groups (P-value = 0.014, P=0.002, respectively). Regarding delayed onset of neovascularization, there was a significant difference in the timolol and bevacizumab group in rabbits (P-value = 0.04, P=0.00, respectively). In conclusion, the use of timolol and bevacizumab drops besides betamethasone can delay neovascularization and decrease the length of corneal vascularization in rabbits.

## INTRODUCTION

The cornea is a clear and avascular structure essential to create a proper anterior refractive surface [1]. Corneal disease is the third most common cause of impaired vision worldwide. Although the prevalence and incidence of corneal neovascularization (CNV) are unknown globally, it is predictable that 1.4 million people develop CNV yearly, and vision loss occurs in 12% of them [2]. CNV is caused by a variety of etiologies, such as hereditary diseases, contact lens hypoxia, inflammatory conditions, chemical burns, stem cell deficit, allergies, ocular trauma, infectious keratitis, autoimmune diseases and corneal transplant rejection [3]. Neovascularization of the cornea involves sprouting of new vessels, especially of the capillaries and venules around the cornea. Development of new and uncontrolled vessels in the eye is an important process in the pathogenesis of various types of eye diseases including Herpes virus keratitis, diabetic retinopathy, and age-related macular degeneration, as well as chemical and physical injuries [4]. 

In the pathogenesis of CNV, the balance between proangiogenic and anti-angiogenic factors is disrupted, which induces expression of Vascular endothelial growth factor (VEGF) [5]. The VEGF factor and its dependent molecules seem to play a key role in signal regeneration of the neovascularization process [6]. Various medications or surgeries are used in the management of CNV. There are several methods available for the management of CNV including corticosteroids, cyclosporine, bevacizumab, ranibizumab, non-steroidal anti-inflammatory drugs, systemic methotrexate and thalidomide [7]. These treatments have side effects and sometimes patients are not satisfied with these medications. In the recent years, there has been a great interest in the management of CNV by anti-VEGF (drops, subconjunctival and intra-stromal) [2]. Bevacizumab (monoclonal anti-VEGF) is commonly used for the treatment of CNV pre- and postoperatively [8]. Bevacizumab is widely used for the treatment of various diseases such as colorectal cancers. Intravitreal injection of bevacizumab is used in the treatment of macular degeneration, choroidal neovascularization and diabetic retinopathy [9, 10]. Early injection of bevacizumab may inhibit CNV, while late injection of bevacizumab may not alter macrophage infiltration and cannot prevent expression of VEGF, VEGFR1 and VEGFR2 on corneal vessels [11].

Propranolol is a non-selective beta-adrenergic receptor blocker commonly used in cardiovascular diseases such as ventricular tachyarrhythmia and hypertension. In some studies, propranolol has been shown to have anti-angiogenic and vasoconstrictive effects by inhibiting vascular factors such as VEGF and Fibroblastic growth factor (FGF), making it a target for treatment and control of retinal neovascular diseases [12]. Due to presence of VEGF receptors on the surface of corneal endothelial cells, the possibility of drug effects on corneal endothelial cells has been suggested [13]. Timolol and propranolol are similar in some aspects as both of them are non-selective beta-adrenergic antagonists. The mechanism by which these medicines act on neovascularization regression is mainly indefinite. Nevertheless, these medicines are thought to act over VEGF signaling, vasoconstriction and vascular endothelial cell apoptosis [14, 15]. 

Steroids are commonly used in ophthalmology as a potent anti-inflammatory agent. They are mainly inhibited by phospholipase enzyme and thus by the arachidonic acid pathway (reduced production of prostaglandins). Some prostaglandins exert indirect anticoagulant effects [16]. Some previous studies on CNV have been performed using propranolol, timolol and bevacizumab [12, 15, 17, 18]. Owing to pivotal role of corneal transparency and avascularity in optimal visual quality, this study aimed to compare the effect of propranolol, timolol and bevacizumab with betamethasone in prevention of CNV in rabbits.

## METHODS

This experimental animal study was performed on 28 male rabbits weighing 1500-1900 grams and aged one year approximately (Animal Care Center of Ahvaz Jundishapur University of Medical Sciences). Animals were kept individually in standard cages at optimal room temperature with appropriate ventilation and fed with pellet feed and water for a week to adapt to the new environment. They were kept under twelve hours of light and twelve hours of darkness. The animal's right eye selected for the study. All tenets of the Declaration of Helsinki regarding animal studies were considered. The study was approved by the Ethics Committee of Ahvaz Jundishapur University of Medical Sciences (Code, IORC-9702). 

At first, animals were sedated using intramuscular injection of ketamine (Ketalar; Par Pharmaceutical Co, Inc., The USA) (20mg/kg) and xylazine (Xylazine; Pantex Holland B.V) (2mg/kg). The evaluation was performed by an ophthalmology resident using a slit lamp (Topcon SL-5D; Kogaku Kikai K. K., Tokyo, Japan). Induction of CNV was performed by three loose vertical sutures with 7-0 silk (Supasil, Supa Medical Devices, Iran) approximately 2 mm long and 1 mm distal to the limbus in three areas. All surgeries were performed by one person (MS.M.). Ciprofloxacin eye drops 0.3% (Ciplex; Sina Darou Laboratories Co) were used every six hours for 2 weeks to prevent ocular infection. Rabbits were randomly divided into 4 groups including control group (betamethasone 0.1%, 3 times daily) (Betasonate; Sina Darou Laboratories Co), propranolol (Pranol; Tolid Daru Co) group (betamethasone 0.1%, 3 times daily + propranolol 10mg/mL, 3 times daily), timolol group (betamethasone 0.1% drops 3 times daily + timolol 0.5% drops, 3 times daily) (Timolol; Sina Darou Laboratories Co) and bevacizumab (Avastin; Genetech, Inc.) group (betamethasone 0.1% drops 3 times daily + bevacizumab drops 4 mg/mL, 2 times daily) and continued until initiation of neovascularization up to 6 weeks. Rabbits were examined on 7^th^, 14^th^, 21^st^, 28^th^, 35^th^ and 42^nd^ days. In each examination photography was performed using a CANON Camera (5D, MARK3, JAPAN). Progression of intra-corneal vessels was assessed in each examination. The length of vascular advancement (the closest distance from the vessels to the limbus) (millimeter; mm) and the level of corneal vascular area (square millimeter; mm^2^) (corneal area with vessels) were measured with the help of a Cursor. All images were evaluated using ImageJ software version 1.49 (Image processing, NIH, Bethesda, Maryland). 

Descriptive statistics were used for analysis, including mean and standard deviation (SD) for quantitative variables and relative percentages and frequencies for qualitative variables. A Chi-square test was used to assess the association between quantitative variables. Data analysis was performed using SPSS 22 software (SPSS Inc., IBM Corporation., Platform Java, Type: statistical analysis).

## RESULTS

**Figure 1:**
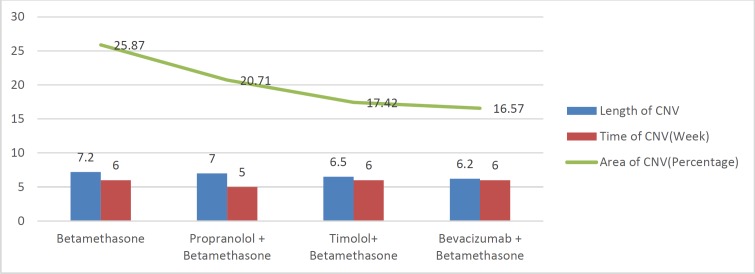
Actual Mean of Vascular Progression, the Time of Neovascularization and Vascular Area in Four Study Groups. Abbreviations: CNV: Corneal Neovascularization; Length of CNV: Mean in millimeter.

**Figure 2:**
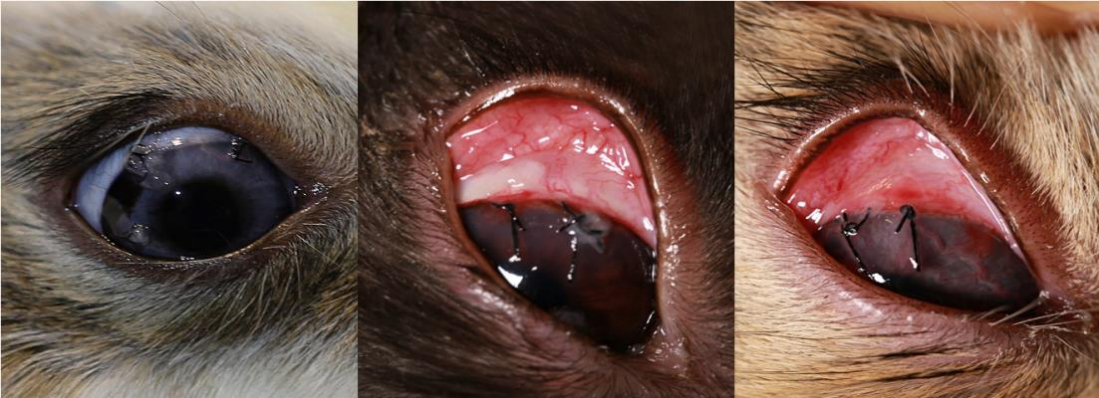
Control Group: 7th, 21st and 42nd Days of Study; Significant Progression of Area and Length of corneal neovascularization (CNV) in 42nd Day of Study.

**Figure 3:**
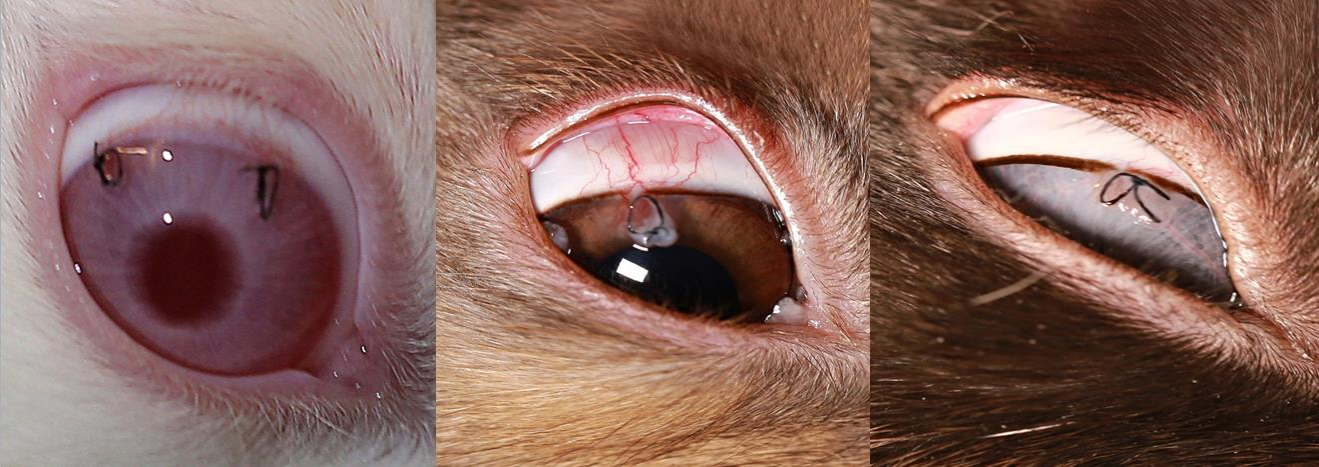
Propranolol Group: 7th, 21st and 42nd Days of Study; Progression of Area and Length of corneal neovascularization (CNV) in 42nd Day of Study Compared to the Control Group.

**Figure 4:**
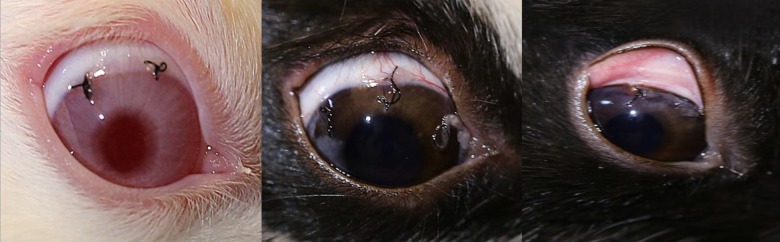
Timolol group: 7th, 21st and 42nd Days of Study; Significant Less Progression of Area and Length of corneal neovascularization (CNV) in 42nd Day of Study Compared to the Control and Propranolol Groups.

**Figure 5:**
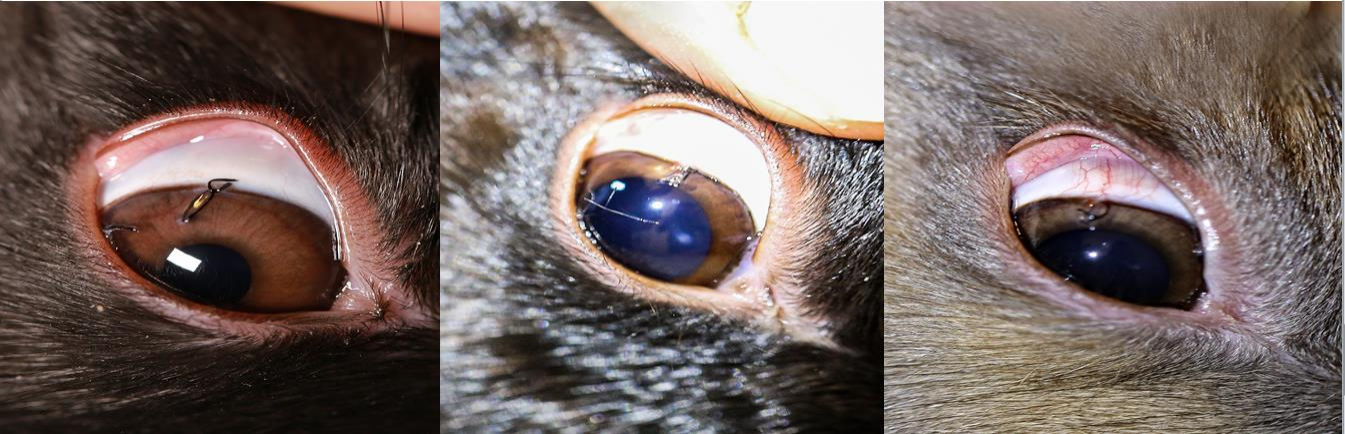
Bevacizumab Group: 7th, 21st and 42nd Days of Study; Significant Less Progression of Area and Length of CNV in 42nd Day of Study Compared to Control and Propranolol Groups

CNV occurred in the study groups at different weeks. Figure 1 shows actual mean of vascular progression, the time of neovascularization and vascular area in all four groups. At the end of study, vascular progression, the time of neovascularization and vascular area were evaluated in all the four groups compared to the control group (betamethasone alone). All the three variables (area, length, and time) were recorded in all four groups and compared with each other (Table 1). In this study, the area of ​​vessels in the propranolol + betamethasone, timolol +betamethasone, and bevacizumab + betamethasone groups were compared to the control group (betamethasone alone). There was a significant difference between the three study groups and the control group regarding reduction in the area of neovascularization in rabbits, which showed a significant decrease in the timolol and bevacizumab groups (Table 2) (P-value = 0.05, 0.047). On the 42^nd^ day of study, comparison of the study groups regarding the length of neovascularization showed significantly less progression in timolol and bevacizumab study groups (P-value=0.014, P-value=0.002) (Table 3). Regarding delayed onset neovascularization in the three study groups compared to the control group (betamethasone alone), there was a significant difference between the propranolol, timolol and bevacizumab groups compared to the control group (P-value = 0.04, 0.00, and 0.00, respectively) (Table 4). Figures 2**, **3**, **4**, **5 show the progression and area of CNV in the study groups at 7^th^, 21^st^ and 42^nd^ days of study. In the last photo of each figure (42^nd ^day) specifically in the control group, a dominant increase of length and area of CNV is seen. 

**Table 1:** Mean Rank of Three Variables of Corneal Neovascularization in the Four Study Groups

	Groups	Number	Mean Rank
Time; days	Betamethasone	7	5.64
	Propranolol + Betamethasone	7	12.50
	Timolol + Betamethasone	7	20.64
	Bevacizumab + Betamethasone	7	19.21
	Total	28	
Area; square millimeter	Betamethasone	7	23.64
	Propranolol + Betamethasone	7	15.64
	Timolol + Betamethasone	7	10.07
	Bevacizumab + Betamethasone	7	8.64
	Total	28	
Length; millimeter	Betamethasone	7	21.29
	Propranolol + Betamethasone	7	18.29
	Timolol + Betamethasone	7	10.50
	Bevacizumab + Betamethasone	7	7.93
	Total	28	

**Table 2:** Reduction in the Area of Corneal Neovascularization (percentage) in the Three Study Groups Compared to the Control Group.

(I) groups	(II) groups	Mean Difference	p-value	95% Confidence Interval	
				Lower Bound	Upper Bound
Betamethasone	Propranolol + Betamethasone	5.14	0.085	-3.63	13.91
	Timolol + Betamethasone	8.71	0.050	-0.06	17.49
	Bevacizumab + Betamethasone	9.29	**0.047**	0.51	18.06

**P value less than 0.05 in bold.**

**Table 3:** Length of Corneal Neovascularization (millimeter) in the Three Study Groups Compared to the Control Group.

(I) groups	(II) groups	Mean Difference	p-value	95% Confidence Interval	
				**Lower Bound**	**Upper Bound**
Betamethasone	**Propranolol + Betamethasone**	-0.86	**0.04**	-1.71	0
	**Timolol + Betamethasone**	-1.71	**0.00**	-2.57	-0.86
	**Bevacizumab + Betamethasone**	-1.57	**0.00**	-2.42	-0.72

**P value less than 0.05 in bold.**

**Table 4:** Delayed Onset Corneal Neovascularization in the Three Study Groups Compared to the Control Group.

(I) groups	(II) groups	Mean Difference	p-value	95% Confidence Interval	
				Lower Bound	Upper Bound
Betamethasone	Propranolol + Betamethasone	0.25	0.67	-0.37	0.88
	Timolol + Betamethasone	0.75	**0.014**	0.12	1.38
	Bevacizumab + Betamethasone	0.92	**0.002**	0.29	1.55

**P value less than 0.05 in bold.**

## DISCUSSION

There was a significant decrease in the area of neovascularization in the timolol and bevacizumab groups (P-value = 0.05, and 0.047, respectively). Regarding length of CNV, there was a significantly less progression in the timolol and bevacizumab groups (P-value = 0.014, and 0.002, respectively). Regarding delayed onset of CNV, a significant difference in the propranolol, timolol and bevacizumab group was observed (P-value = 0.04, 0.00, and 0.00, respectively). 

Imbalance in angiogenesis is an underlying mechanism for neovascularization. Vascular endothelial growth factor is one of the elements associated with neovascularization. Many VEGF inhibitors have been used for retinal and some corneal disorders. One of important VEGF inhibitors is bevacizumab. Because of the efficiency of bevacizumab as an anti-VEGF, it has been considered as a potential management choice for neovascularization on the cornea [19]. Bevacizumab prevents interaction between VEGF-A and its receptors (VEGFR-1 and VEGFR-2). Rocher et al. found a comparable result to our study in gerbils. Compared to the control group, subconjunctival injection of a 150 kDa rat anti-VEGF antibody (0.02 mL 10µg/mL) reduced the neovascularization area by more than 20%, while topical application (10µg/mL) reduced it by 15% [20]. Given the presence of VEGF receptors on the surface of corneal endothelial cells, the possibility of drug effects on corneal endothelial cells has been suggested [21, 22]. Luis de Redin et al. evaluated the effect of bevacizumab-loaded albumin nanoparticles in a rat model of CNV. Results established by histopathological analysis clearly revealed that eyes treated with nanoparticles displayed lower levels of fibrosis, inflammation and edema [23]. Baradaran-Rafii A et al. conducted a study to compare Adiponectin versus bevacizumab eye drops for the treatment of CNV during 14 days. Results showed reduction of CNV in both groups but more in Adiponectin [24]. Sahan B et al. compared the inhibitory effects of dovitinib and bevacizumab for the treatment of CNV in a rat model and showed a statistically significant decrease in the percentage of CNV in all the treatment groups, which was not statistically significant in the percentage of CNV between bevacizumab and bevacizumab +dovitinib groups [25]. Study of Xu X et al. on prevention of CNV by subconjunctival injection of bevacizumab loaded thermosensitive hydrogels in rabbits confirmed the effect of bevacizumab in the treatment of CNV. They also suggested that proposed Ava-PECE hydrogel might be a capable vehicle for the treatment of CNV [26]. Lopes GJA et al. showed the inhibitory effect of topical application and subconjunctival injection of bevacizumab without any significant difference on CNV in rabbits after chemical burning of the cornea [27]. Huang J et al. concluded that combined Dexamethasone and Avastin (bevacizumab) by supramolecular hydrogel exhibited an advantage over Avastin monotherapy approach, which might be a promising alternative therapy for inflammatory CNV [28], which is similar to our results as we used combined betamethasone and bevacizumab. Sella R et al. and Gal-Or O et al. conducted compared the efficacy of topical and Subconjunctival Aflibercept with Bevacizumab for the prevention of CNV in a rat model. They concluded that aflibercept effectively inhibits CNV in a rat model of chemical burn-induced neovascularization and has more efficacy compared to bevacizumab [29, 33]. These two studies confirmed the effect of anti-VEGF agents in the treatment of CNV, but on the other side showed a less effect of bevacizumab. Moreover, Ozdemir O et al. revealed that both subconjunctival and topical administrations of bevacizumab prevent CNV and reduce inflammation and fibroblast activity in a rat model. Also, they did not find significant differences between subconjunctival injection and topical administration of bevacizumab [30], which is consistent with a meta-analysis performed by Papathanassiou M et al. They concluded that both topical and subconjunctival bevacizumab accomplish a significant decrease in the area of CNV [31]. In this study, CNV was induced by alkali burn, but we used corneal suturing to induce the CNV. Our study was in the line with abovementioned studies in part which showed the effect of anti-VEGF agents, specifically bevacizumab, in the treatment of CNV. 

Side effects of timolol include blinking, burning, staining, itching, redness or pain in the eye and headaches [32]. Propranolol with its anti-vascularization and vasoconstriction effects and also inhibition of VEGF and FGF might control retinal neovascular diseases [34]. However, Filippi L et al. reported two patients with CNV due to Stevens-johnson syndrome complications, who did not respond to steroids and cyclosporine well. They showed a positive effect of propranolol eye drops in the treatment of CNV, which is in line with the present study [35]. The results of our study can provide useful information for effective improvement or prevention of CNV. In the study of Simavli et al. in 24 eyes of 24 mice, neovascularization of the cornea was induced using NaOH. They showed that the mean percentage of CNV in the first group (treated with normal saline drops) was 59%, in the second group (treated with dexamethasone drops) 25.5%, in the third group (treated with propranolol 1 mg/mL drops) 68.9% and in the fourth group (treated with propranolol 0.5 mg/mL drops) 50.4%. There was no significant difference between the third and fourth groups with the first group. Whereas the second group had significantly less CNV area than the other groups. They concluded that topical propranolol at a dose of 1 and 0.5mg/mL did not affect NaOH-induced CNV in mice [12]. Similar to the present study, no significant reduction in neovascularization after treatment with propranolol was observed in mice, which confirms the results of our study. However, propranolol combined with betamethasone eye drop delayed CNV onset compared with betamethasone eye drop alone. 

Kasiri et al. conducted a study to inhibit CNV by timolol in the rabbit model. Neovascularization was induced in the eyes of 20 rabbits and then divided into two groups; the timolol group received 0.5% timolol eye drops twice daily, and the control group received saline drops two times per day for two weeks. The mean CNV area after 7 days was significantly lower in the timolol group than the saline group (P <0.001). After 2 weeks, the mean CNV in the timolol group were 1.33 ± 0.85 and 2.06 ±1.73, correspondingly (P = 0.315). After the first week of treatment, timolol significantly reduced CNV compared to controls [15]. which is consistent with the current study regarding the efficacy of timolol in improvement and prevention of CNV. 

Furthermore, Öner et al. randomly divided 20 rats with silver nitrate induced CNV into four groups. The control group received artificial tears twice daily, the second group received subconjuctival bevacizumab in the first, fourth and seventh days, and the other two groups received topical bevacizumab at doses of 4 and 12.5 mg/mL twice weekly. The results showed that the mean percentage of CNV was 63% in the control group, 30% in the injected bevacizumab group and 27% and 26% in the other two groups. In this study, a significant difference was observed between the control and other groups (p <0.01). They concluded that subconjunctival injection and topical administration of bevacizumab are effective and safe in the control of CNV [17]. It is consistent with our results which showed that bevacizumab and timolol were effective in decreasing neovascularization area (P-value: 0.05,0.047) and decreasing neovascularization length (P-value: 0.014, 0.002) compared to betamethasone as a control group. 

In a case-series study on 5 patients by Felix Bock to evaluate the effect of bevacizumab on CNV, patients with invasive CNV were treated with conventional bevacizumab eye drops (0.5 mg/mL) for 6 months. In this study, neovascularization decreased significantly in all patients with bevacizumab, which is consistent with our study [18]. Moreover, Mohammad Dastjerdi evaluated the efficacy and safety of bevacizumab to treat CNV. He evaluated 10 eyes of 10 patients and followed them for 6 months. Their results showed safety and efficacy of bevacizumab in reducing CNV, which is similar to ours [8]. 

One of the innovations of this study was the use of medications concomitantly with betamethasone, to compare the efficacy of these drugs (propranolol, timolol and bevacizumab drops) with the control group (betamethasone alone). Other innovations in this study were the timing of drug administration at baseline as well as not removing sutures as an angiogenic stimulatory factor. We had some limitations. This study was performed with a relatively small number of laboratory animals. Other limitations of this study include failure to evaluate antibiotic susceptibility and drug resistance, failure to evaluate side effects, and severity of symptoms. Further clinical trials may indicate the efficacy of these drugs in human more clearly. Therefore, further controlled studies are needed to understand the effects of propranolol, timolol and bevacizumab eye drops in the treatment of CNV.

## CONCLUSION

Timolol and bevacizumab eye drops could delay and inhibit neovascularization and decrease the length of CNV in rabbits. Further controlled studies are needed to understand the effects of propranolol, timolol and bevacizumab eye drops in the treatment of CNV.
